# Supplementary material for: Characterization of the Molecular Diversity and Degranulation Activity of Mastoparan Family Peptides from Wasp Venoms
Source: Toxins (Basel). 2023 May 12;15(5):331. doi: 10.3390/toxins15050331 (PMC10222454; doi:10.3390/toxins15050331)
Supplement: Supplementary file 1 [file toxins-15-00331-s001.zip › toxins-2330884-supplementary.pdf]

Supplementary Materials

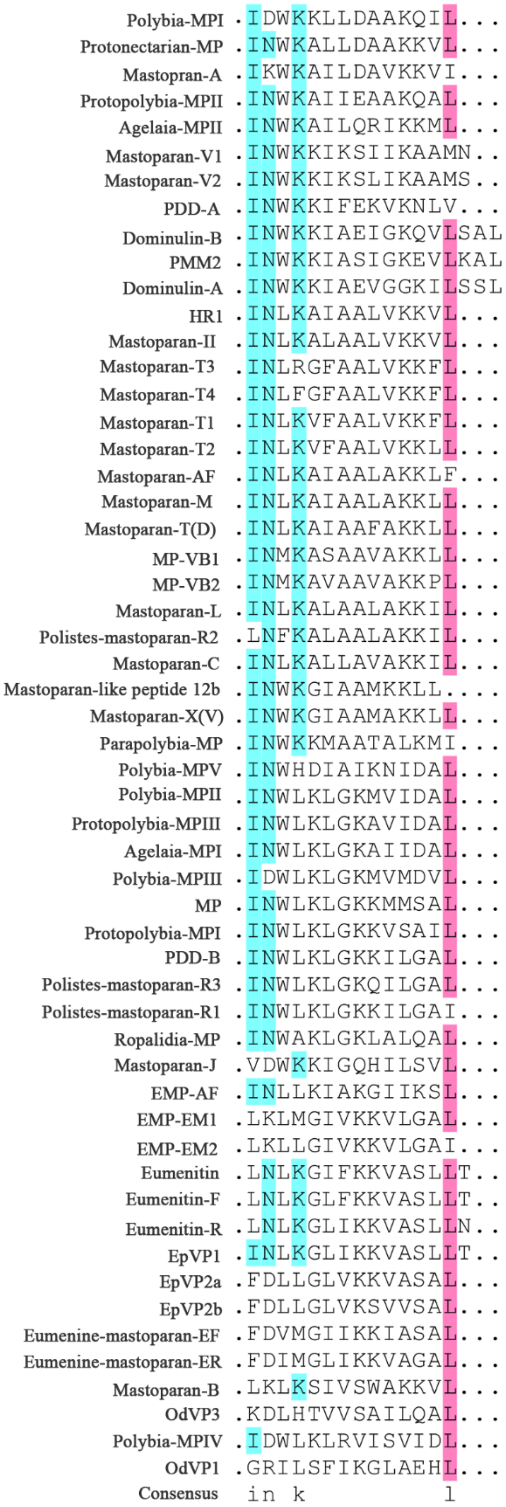

Figure S1. Sequence alignment of 55 wasp venom mastoparans. Amino acid residues were marked with, pink (>75% identity), light blue (>50% identity) and blank (< 50% identity).
